# Notes and News

**Published:** 1905-06-24

**Authors:** 


### Notes and News.

A matinee concert in aid of the League of Mercy will be given, under the patronage of Princess Alexander of Teck, on June 27, at Grosvenor House.

The late Mr. James Burrows, of Nottingham, has bequeathed the residue of his property, estimated at about £10,000, to the Nottingham General Hospital, the Midland Eye Hospital, and the Nottingham General Dispensary.

Mr. Charles Burt has tendered his resignation of the post of chairman to the Royal Free Hospital, which he has held for the past 10 years. Mr. C. W. Blackman has been appointed as his successor. Mr. Burt still retains the post of treasurer to the hospital.

A new nurses' home for district nurses has been opened at Silvertown. It is a memorial to the late Sir Henry Tate, and is the gift of Lady Tate. It has accommodation for five nurses, who are each provided with a bedroom. There is also a sitting-room, dining-room, district-room, changing-room, bath and box-rooms.

The Local Government Board are amending the order relating to the certificate of proficiency in vaccination which is required to be produced by a medical practitioner before he can enter into a contract with a Board of Guardians for public vaccination. The present requirements of the Article of the vaccination order entail some hardship on those who took a degree before the vaccination order was in force, and the Local Government Board will now not require a fresh course of instruction to be undertaken by any registered medical practitioner who possesses a diploma, license, or degree conferring a right of registration under the Medical Acts granted (*a*) by an examining body in England and Wales or Scotland before the date on which that body first required the prescribed certificate as a condition of obtaining the diploma, license, or degree; and (*b*) by an examining body in Ireland before January 1, 1906.

Dr. E. Stirling's subject for the Croonian lectures is "The Chemical Correlation of the Functions of the Body." Dr. F. Roberts delivered an oration on Wednesday.

A provincial meeting of the Society for the Study of Disease in Children, held at Sheffield, commenced on June 17th. There was an exhibition and demonstration of pathological specimens and clinical cases. A large number of papers were read and a discussion followed.

The new Infectious Hospital for Dewsbury and district is now completed. It consists of three pavilions and an administration block, with the usual adjuncts. The large pavilions each contain 30 beds, and the isolation pavilion eight beds. Each pavilion is subdivided. The cost is estimated at £29,000.

University College Hospital are issuing a special appeal for funds to complete the building of the new hospital. The generous intention of the late Sir J. Blundell Maple was never fully carried out. The Committee are therefore compelled to provide a sum of about £3,200 for the execution of absolutely necessary structural work.

Amongst the buildings damaged by the recent earthquake in India was the hospital at Karnal, which is conducted by the Cambridge Mission at Delhi. The hospital was only a rented building and the owner will not rebuild. The work of the hospital is consequently reduced to a dispensary. The mission appeals for £2,000 to build a hospital which is much needed.

Mr. E. A. Attwood has been promoted to the post of secretary of the Homœopathic Hospital in Great Ormond Street. He has been assistant secretary for 22 years, and is thoroughly conversant with the working of the institution. The Homœopathic Hospital has received a legacy of £100 from the late Mr. James Epps, jun., son of Mr. James Epps, cocoa manufacturer.

We are glad to learn that the Saltaire Hospital is to be extended by new buildings added to the present premises. A proposal was made to adapt an existing house into a larger hospital, instead of commencing building operations, but the difficulty of adapting a private residence to hospital requirements fortunately was realised, and the scheme abandoned. An anonymous donation of £1,000 has been received by the hospital, and Messrs. J. S. Morgan have sent a donation of £100.

No less than 15 van loads of pulped fruit put up in barrels and tin cases were recently condemned by the Sanitary Inspectors of Stepney. The fruit was on the premises of the Eureka Preserving Company, Bow. It was in a decomposed and putrid state. It is a matter for congratulation that through the vigilance of the sanitary authorities this particular consignment of unwholesome food did not find its way to the market, but such instances must raise a feeling of apprehension, as it cannot always be possible to detect every misdemeanour of the kind.

